# The Effects of Bacterial Lipopolysaccharide (LPS) on Turkey Poults: Assessment of Biochemical Parameters and Histopathological Changes

**DOI:** 10.3390/vetsci9050240

**Published:** 2022-05-19

**Authors:** Mohamed F. Abou Elazab, Nasr E. Nasr, Mohamed S. Ahmed, Barakat M. Alrashdi, Naief Dahran, Mohamed A. Alblihed, Ehab Kotb Elmahallawy

**Affiliations:** 1 Clinical Pathology Department, Faculty of Veterinary Medicine, Kafrelsheikh University, Kafr El-Sheikh 33516, Egypt; 2 Department of Biochemistry and Clinical Biochemistry, Faculty of Veterinary Medicine, Kafrelsheikh University, Kafr El-Sheikh 33516, Egypt; nasr.nasr@vet.kfs.edu.eg; 3 Pathology Department, Faculty of Veterinary Medicine, Kafrelsheikh University, Kafr El-Sheikh 33516, Egypt; mohamed.abdelrahman1@vet.kfs.edu.eg; 4 Biology Department, College of Science, Jouf University, Sakaka 72388, Saudi Arabia; bmalrashdi@ju.edu.sa; 5 Department of Anatomy, Faculty of Medicine, University of Jeddah, Jeddah 21959, Saudi Arabia; ndahran@uj.edu.sa; 6 Department of Microbiology, College of Medicine, Taif University, P.O. Box 11099, Taif 21944, Saudi Arabia; mabulihd@tu.edu.sa; 7 Department of Zoonoses, Faculty of Veterinary Medicine, Sohag University, Sohag 82524, Egypt

**Keywords:** LPS, turkey poults, electrophoresis, serum biomarkers, pathology

## Abstract

A lipopolysaccharide (LPS) is a large molecule and an outer membrane glycolipid found in Gram-negative bacteria, including *Escherichia coli* (*E. coli*). These molecules (LPS) target acute inflammatory responses and significant physiological changes. Importantly, *E. coli* is considered one of the most important bacterial causes of avian colibacillosis that affect domestic turkey industry. However, little information is available about the potential influence of LPS on the biochemical parameters and histopathological changes in turkey poults. Therefore, this study aimed to evaluate the influence of bacterial lipopolysaccharide (LPS) molecules on serum biomarkers and histopathological changes in turkey poults. The birds were randomly divided into five groups, as follows: group I did not receive any inoculation; group II was inoculated with sterile saline; and groups III, IV, and V were inoculated intraperitoneally with LPS at 0.01, 0.1, and 1 mg/kg of body weight (BW), respectively. The biochemical parameters and the histopathology of different organs were examined in all birds one day post-inoculation. Our results revealed hypolipidemia, hypoglycemia, a significant decrease in uric acid, and a significant increase in serum activities of aspartate transaminase (AST), alanine transaminase (ALT), alkaline phosphatase (ALP), lactate dehydrogenase (LDH), and creatine kinase (CK), as well as cardiac troponin T concentrations in treated groups. Moreover, there was a significant increase in α1-, β-, and γ-globulin concentrations and a decrease in albumin and α2-globulin concentrations in group V. However, a significant increase in α2- and γ-globulin levels and a decrease in albumin levels were detected in groups III and IV. In addition, significant decreases in the albumin/globulin ratio were recorded in all LPS-treated groups. Hepatocellular and cardiac muscle necrosis, slight renal changes, and massive pulmonary inflammatory reactions were recorded. This study provides valuable information about serum biomarkers, protein fractions, and histopathological changes in turkey poults treated with LPS for further investigations of pathophysiological mechanisms in avian medicine along with biomedical research.

## 1. Introduction

The poultry industry is one of the fastest-growing sectors of the animal industry in Egypt. Among others, turkey is considered an important domesticated poultry species in Egypt raised for meat production. However, similarly to many countries, the turkey production system in Egypt is susceptible to various pathogens, which results in serious infections that hinder this industry. These harmful agents include bacteria, viruses, and parasites that cause severe economic losses and create a challenge for poultry producers. Among these bacterial diseases, avian colibacillosis is considered the most widespread bacterial infection, which causes significant economic losses to the poultry industry worldwide [1]. In turkey, colibacillosis is also considered one of the major bacterial diseases affecting the domestic turkey production system [2]. This infectious disease is caused by the pathogenic strain of *Escherichia coli* (*E. coli*), which is considered the most abundant disease-producing strain in domestic turkey, leading to reduction in egg and meat production and ultimately resulting in high mortalities [3]. Avian colibacillosis, caused by *E. coli*, might result in a series of serious clinical manifestations in turkeys, including cellulitis, colisepticemia, omphalitis, osteomyelitis, peritonitis, swollen head syndrome, synovitis, panophthalmitis, salpingitis, and coligranuloma, besides being frequently fatal [4,5]. It is therefore not surprising to mention that some previous reports used turkey as animal model for better understanding avian colibacillosis [6,7,8,9,10].

It is noteworthy to state that lipopolysaccharide (LPS) or endotoxin is considered a principal constituent of the outer leaflet of the outer membrane of Gram-negative bacteria, such as *E. coli* and *Salmonella typhimurium*. This unique glycolipid component triggers the abandoned production of inflammatory mediators and targets oxidative stress, which in turn may result in septic shock, multiple organ dysfunction and failure [11]. LPS is an important factor that causes cell and tissue injury, and it induces multiple organ dysfunction and various histopathological changes [12,13]. LPS molecules also interact with other biological systems through their involvement in host–pathogen interactions and a series of critical events necessary for bacterial growth and multiplication, such as adhesion, colonization, virulence, and symbiosis. It is therefore not surprising to mention that LPS is considered a potent elicitor of innate immune response and serves as an early warning signal of bacterial infections [14]. Taken into account, the resulting endotoxemia and sepsis can result in a series of health complications, such as myocardial dysfunction, acute respiratory failure, and multiple organ failure [15]. Furthermore, during sepsis, a life-threatening and multiple organ failure together with the moderately regulated immunoinflammatory response can equip the causative agent with an effective defense against pathogenic microorganisms and, consequently, mild tissue damage commonly leads to multiple organ dysfunction or secondary infection, whereas a severe immunoinflammatory dysfunction, especially an excessive pro-inflammatory dysfunction or immunosuppression [16]. Taken into account, several clinical studies have shown that the level of circulating LPS is correlated with disease severity, the onset and extent of organ dysfunction, and mortality [17]. As mentioned above, turkey occupies a significant role next to chicken and duck in augmenting the economic and nutritional status of various populations worldwide, but *E. coli* is the most abundant disease-producing strain in turkey. It should be taken into account that evaluation of serum proteins and their electrophoretic patterns combined with assessment of the histopathological changes have been considered well-established diagnostic tools for the detection and monitoring of various diseases in veterinary medicine. Revising the available literature, there is limited knowledge as to the effect of stress exposure on different biological markers and the histopathological effects of LPS effects on turkey poults. Given the above information, this study aimed to evaluate the influence of bacterial LPS administration as a stressor on various serum biomarkers and protein profiles and the histopathology of turkey poults. Furthermore, this study examined the effect of stress exposure on different biological markers.

## 2. Materials and Methods

### 2.1. Ethical Considerations

This study was approved by the Research Ethics Committee of Faculty of Veterinary Medicine, Kafrelsheikh University, and the Institutional Review Board number was KFS-2021/03.

### 2.2. Animals

A total of fifty turkey male poults (one day old) were obtained from a commercial hatchery. The birds were maintained in a controlled environment and provided with food and chlorinated water *ad libitum*. This experiment was performed in accordance with national/institutional guidelines for the care and use of animals, and all precautions were taken to prevent any kind of cruelty.

### 2.3. Chemicals

Ultrapure LPS solution from *E. coli* serotype 055: B5 (UP LPS, tlrl-peklp), purified by phenol extraction, was provided by Sigma Chemical Inc., St Louis, MO, USA, then dissolved in sterile 0.9% NaCl solution (1 mg LPS/mL saline). All chemicals used in this experiment were of grade 1.

### 2.4. Experimental Design

A total of 50 five-week-old male turkey poults, weighing 439.25 ± 62.88 g, were randomly divided into 5 groups of 10. The first group I did not receive any inoculation and was considered the negative control group. Birds in the other groups (II, III, IV, and V) received either normal saline or LPS by intraperitoneal injection once, as follows: the birds in group II were injected with normal saline (the control group), those in group III with LPS (0.01 mg/kg body weight (BW)), those in group IV with LPS (0.1 mg/kg BW), and those in group V with LPS (1 mg/kg BW). The logarithmic concentrations used to evaluate the effect of LPS in turkey poults were based on the available literature [18,19,20,21,22,23].

### 2.5. Blood Sample Collection

Blood samples were collected from the wing vein of all birds 24 h after administration of LPS using a 3 mL syringe with a 25 G needle. The blood samples were placed in glass tubes without anticoagulant and kept at room temperature for 8 h. Serum was separated from the coagulated blood using centrifugation at 3000 rpm for 15 min and then stored at −20 °C until use.

### 2.6. Serum Biochemical Analysis

In this step, serum total protein, albumin, triglycerides (TG), total cholesterol (TCh), high-density lipoprotein (HDL), low-density lipoprotein (LDL), glucose, urea and uric acid, creatinine, and uric acid levels; the serum activities of aspartate transaminase (AST), alanine transaminase (ALT), alkaline phosphatase (ALP), lactate dehydrogenase (LDH), and creatine phosphokinase (CK), and cardiac troponin T (cTnT) were determined using an automatic chemical analyzer (Cobas 8000, Roche, Germany).

### 2.7. Serum Protein Electrophoresis

The total serum protein concentration was evaluated calorimetrically. Then, serum proteins were fractionated using electrophoresis on cellulose acetate in a Serum Electrophoresis Automated System (Minilite, Cell Diagnostic Division, Milan, Italy). Absolute values (g/dL) for each protein fraction were measured by multiplying the percentages of each fraction by the total protein concentrations. Five protein fractions (albumins, α1-globulins, α2-globulins, β-globulins, and γ-globulins) were identified and assessed in all serum samples. The absolute values for each fraction were mathematically obtained by multiplying the percentage by the total protein concentration. The albumin/globulin (A/G) ratios were calculated by dividing serum albumin concentrations by the sum of the serum globulin concentrations individually.

### 2.8. Histopathological Assessment

After scarification of all birds, postmortem examinations were performed, and all observed lesions were recorded. Specimens from all organs, mainly the liver, heart, kidneys, and lungs, were also fixed in 10% neutral phosphate-buffered formalin (pH 7.2) for histopathological examination. The specimens were then dehydrated in ascending grades of alcohol, cleared in xylene, embedded in paraffin wax, sectioned (4 μm thickness), then stained with hematoxylin and eosin (HE) and examined using light microscopy [24]. Furthermore, the reported histopathological lesions were scored according to set criteria: marked (massive) (41–100% of 188 tissue involved), moderate (21–40% of tissue involved), mild (slight) (11–20% of tissue involved), and minimal (0–10% of tissue involved), in addition to reporting the nature and extent of the lesion and its frequency of occurrence inside the tissue as described elsewhere [25,26].

### 2.9. Statistical Analysis

The data were analyzed using the one-way analysis of variance followed by Tukey’s multiple comparison test. GraphPad Prism 5 software was used to analyze the data. All data were presented as means ± SD for chicks in each group and represent three independent repetitions. The threshold for statistical significance was set at *p* < 0.05, and *p* < 0.005 was considered significant and highly significant, respectively.

## 3. Results

### 3.1. Effects of LPS on Serum Biomarkers

LPS injections, at doses of 0.1 and 1 mg/kg BW (group IV and V), induced significant reductions in the serum levels of triglycerides, cholesterol (TCh, HDL, and LDL), glucose, urea, and uric acid. However, LPS at a dosage of 0.01 mg/kg BW (G III) significantly decreased serum levels of cholesterol (TCh and LDL), glucose, urea, and uric acid only compared to the control groups (*p* < 0.05; Table 1). Conversely, LPS at a dosage of 0.1 and 1 mg/kg BW (groups IV and V) significantly increased the serum activities of AST, ALT, ALP, LDH, and CPK, as well as cTnT concentrations. However, LPS at a dosage of 0.01 mg/kg BW (group III) significantly increased the serum levels of increased serum activities of AST, ALT, ALP, LDH, and CPK only compared to the control groups (*p* < 0.05; Table 1).

### 3.2. Effects of LPS on Serum Protein Fractionation

Serum proteins were separated using cellulose acetate electrophoresis, and five fractions (albumin, α1-, α2-, β-, and γ- globulins) were identified. Significantly higher serum concentrations of α1-, β-, and γ-globulins were observed in group V; however, significantly higher serum concentrations of α2- and γ-globulins were detected in groups III and IV compared with the control groups (*p* < 0.05; Table 2). Conversely, group V showed significant reductions in serum albumin and α2-globulin concentrations, whereas groups III and IV showed significant reductions in albumin concentrations only compared with the control groups (*p* < 0.05; Table 2). Additionally, significant reductions in the mean values of the A/G ratios were recorded in all treated groups when compared with the control groups (*p* < 0.05; Table 2).

### 3.3. Histopathological Examination

**Figure 1:**
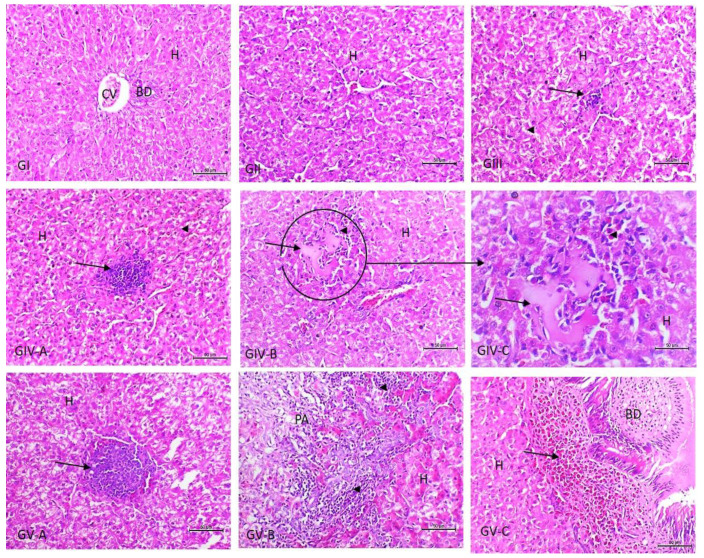
Photomicrograph of liver sections from experimental groups stained with Hx & E stain. G1I and GI1: the liver showing normal architecture, central vein, and intact hepatocytes (H); GIII: there was slight Kupffer cell activation (arrow) and slight vacuolation in the cytoplasm of hepatocyte (arrowhead). GIV-A: moderate Kupffer cell activation (arrow); GIV-B and -C: slight hepatocellular necrosis (arrow) surrounded by a few inflammatory cells (arrowhead); GV-A: a focal collection of a large number of macrophages (granuloma-like) within the hepatic parenchyma (arrow). GV-B: hepatocellular necrosis in the portal area (PA) with infiltration of several chronic inflammatory cells within and around the necrosed areas (arrowhead); GV-C: periductal inflammatory cell infiltration with hyperplasia of the bile duct lining epithelium (arrow) in the PA. The bar size of each picture is included under each picture.

On HE stained sections, there were no obvious histopathological changes in the organs of birds in groups I and II. As shown in Figure 1, the livers of the birds in groups I and II showed no histopathological abnormalities; meanwhile, there was a slight Kupffer cell activation in the livers of birds of group III. However, the livers of birds in group IV showed moderate Kupffer cell activation, cytoplasmic vacuolation, and slight hepatocellular necrosis. The inflammatory reaction was more pronounced in the birds of group V, wherein the necrosis ranged from a granuloma-like formation, with a localized collection of many macrophages within the hepatic parenchyma and in the portal area, to massive hepatic necrosis, with the infiltration of several chronic inflammatory cells within and around the necrosed areas.

**Figure 2:**
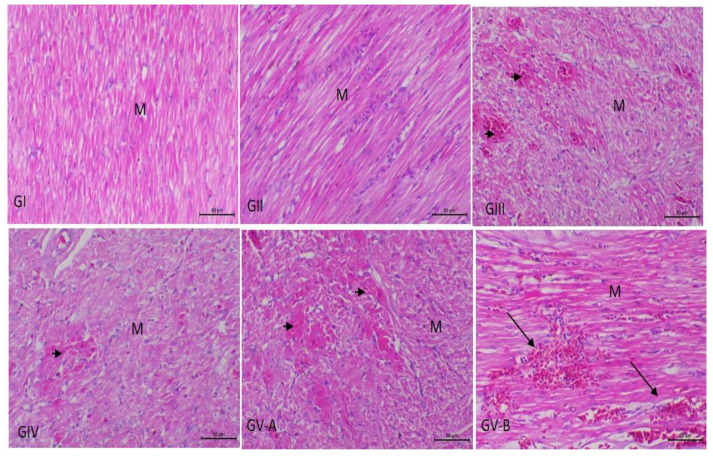
Photomicrograph of heart sections from experimental groups stained with Hx & E stain. GI and GI1: the heart shows a normal arrangement of cardiac muscle fibers (M); GIII and GIV: focal muscle necrosis (arrowhead); GV-A: massive myocardial necrosis (arrowhead); GV-B: myocardial hemorrhage with extravasated RBCs in-between the cardiac muscle fibers (arrows). The bar size of each picture is included under each picture.

**Figure 3:**
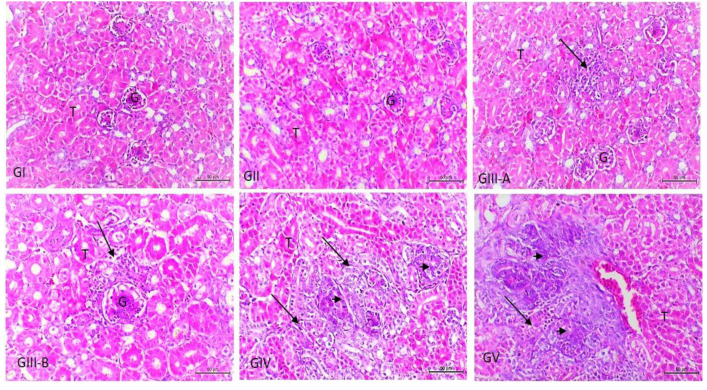
Photomicrograph of kidney sections from experimental groups stained with Hx & E stain. GI and GI1: kidneys show intact glomeruli (G) and renal tubules (T). GIII-(A and B): kidneys show slight interstitial and periglomerular mononuclear cell infiltration (arrow). GIV and GV: there was heavy interstitial and periglomerular mononuclear cell infiltration (arrow) and slight tubular basophilia (arrowhead). The bar size of each picture is included under each picture.

**Figure 4:**
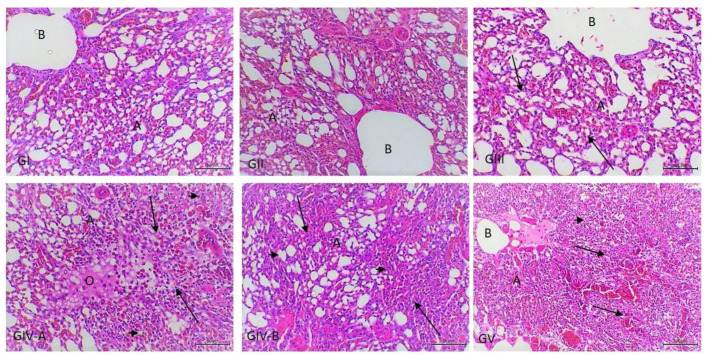
Photomicrograph of lung sections from experimental groups stained with Hx & E stain. GI and GI1: the lungs show normal alveoli (A) and opened airways (B); GIII: slight interstitial polymorphonuclear, eosinophils, and mononuclear cell infiltration (arrow); GIV: thickening of the interalveolar septa with a large number of the inflammatory cells (arrow), edema (O), and the collapse of some alveoli (arrowhead); GV: severe inter- and intra-alveolar infiltration of polymorphonuclear and mononuclear inflammatory cells (arrow) and the collapse of the alveoli and the airways (arrowhead); The bar size of each picture is included under each picture.

In accordance with the heart (Figure 2), cardiac muscles show focal necrosis in groups III and IV. Massive myocardial necroses with the absence of normal muscle striations and myocardial hemorrhages were noticed in group V. Regarding the kidneys, they showed an insignificant inflammatory reaction, only slight mononuclear cell infiltration in the interstitial and periglomerular areas in group III, and slight tubular basophilia in both groups IV and V (Figure 3). As presented in Figure 4, the lungs show slight interstitial polymorphonuclear eosinophils and mononuclear cell infiltration in the birds of group III, thickening of the interalveolar septa with a large number of inflammatory cells, edema, and the collapse of some alveoli in the birds of group IV. Severe inter- and intra-alveolar infiltration of polymorphonuclear and mononuclear inflammatory cells and the collapse of the alveoli and the airways of the birds of group V are shown.

## 4. Discussion

LPS is an endotoxin that commonly used to induce an acute-phase reaction in humans and animals because of its proinflammatory properties [27]. LPS is also known as a potent stimulus that can induce strong immunological and inflammatory responses, which result in extensive tissue damage, in addition to its central role in mediating diseases caused by Gram-negative bacteria [28]. Furthermore, LPS binds to the LPS-binding protein and CD14, and therefore targets the inflammatory response by triggering the production of inflammatory cytokines, including TNF-a, IL-1, and IL-6, via the activation of the TLR4 signaling pathway, leading to multiple organ failure, tissue injury, and death [28,29]. It is noteworthy to state the pathophysiological relationship between endotoxemia and the systemic inflammatory response, and multiorgan dysfunction is not well understood partly because of the lack of suitable animal models [30,31,32]. 

The results of our experiment demonstrated that injection of LPS, especially at doses of 0.1 and 1 mg/kg BW (groups IV and V), induced hepatic damage or dysfunction. The hepatic damage was obvious through significant elevations in the levels of serum transaminases (ALT and AST), LDH, and ALP activities; reduced serum albumin; and morphological changes in the hepatic tissue. These observations agree with the findings of several previous studies that reported the hepatotoxic effects of LPS injections [30,31,32]. Curtis et al. (1980) [33] reported that intravenous injections of endotoxins of *Escherichia coli* serogroups O111 and O78 (2 mg/kg) increased the activities of AST, LDH, and sorbitol dehydrogenase in the plasma of 6–11-week-old chickens during the next 24 h. These changes were attributed to tissue damage effects of endotoxins involving the liver. Another previous study [34] reported that LPS significantly increased the serum levels of ALT, AST, and ALP and decreased serum total protein compared with controls, demonstrating marked liver damage. Additionally, in a mouse model, Jiang et al. (2018) [35] reported that LPS administration induced liver dysfunction, as evidenced by significant increases in the serum AST, ALT, and ALP levels and morphological alterations of the liver.

In the present work, the concentrations of α1-, β-, and γ-globulins were higher, while lower concentrations were observed for albumin and α2-globulin in group V. Conversely, higher serum concentrations of α2- and γ-globulins and lower albumin concentrations were recorded in groups III and IV. It should be stressed that many pathological conditions are often associated with slight or more marked alterations in serum protein patterns, such as shifts in the albumin and globulin concentrations [36,37]. Among others, serum albumin is considered a major negative acute-phase protein (APP). During the acute-phase response of inflammation, the demand of the amino acid for the synthesis of positive APPs is markedly increased, which triggers the reprioritization of hepatic protein synthesis. Consequently, albumin synthesis is downregulated, and amino acids are shifted toward the synthesis of positive APPs [38]. Additionally, serum protein electrophoretograms commonly increase the concentrations of α- and/or β-globulins during acute inflammation; however, a considerable increase in γ-globulin (immunoglobulins) level may also be detected [39]. Since many acute-phase proteins migrate to the α region, the concentrations of α1- and α2-globulins may be elevated in acute inflammatory diseases because of the activation of the host inflammatory responses [40]. Inflammatory diseases and infections may also be accompanied by elevations of the β-fraction because of the increased production of some acute-phase proteins that migrate to this region and may be typical of active hepatitis [36]. The elevation of γ-globulins could usually be considered an indicator of a nonmalignant condition and is mostly caused by reactive and inflammatory processes [40]. Measured A/G radios also revealed significantly lower mean values in all treated groups. A/G ratios automatically decrease due to the decrease in albumin and increase in globulin fractions.

Regarding the metabolic parameters in this study, LPS injections induced hypolipidemia, hypoglycemia, and a significant decrease in uric acid. During acute-phase responses, these alterations might protect the host from further injury and minimize tissue damage. It has been demonstrated that the content of serum triglyceride in endotoxin-poisoned mice decreases slightly 2 h post-intoxication compared with the control group. Thereafter, the level of serum TG in the poisoned mice markedly increased after 12–24 h [41]. However, our experiment revealed a decrease in the serum triglyceride level 24 h after LPS inoculation, especially in groups IV and V, when compared with the control group. Plasma HDL and its major component, apolipoprotein A-I, can protect against LPS-induced endotoxemia and acute tissue damage in a mouse model [42]. Additionally, the injection of endotoxin isolated from a pathogenic avian strain of *Escherichia coli* (1 mg/kg intravenously) accelerated the depletion of liver glycogen and was followed by hypoglycemia in chickens. In addition, changes in the plasma urate and albumin content indicated that glucose was synthesized from amino acids and that albumin was catabolized to provide these amino acids [43]. Acute exposure to LPS can cause transient hypoglycemia and insulin resistance by inhibiting hepatic glucose production through the TLR4, MyD88, and NFκB pathways [44].

Cardiac dysfunction is a frequently occurring severe complication of sepsis [45]. In this study, the modulatory effect of graded doses of LPS on cardiac function was investigated by measuring the serum bioactivities of LDH and CK, as well as cTnT concentrations. LDH and CK are considered important cardiac enzymes that orchestrate ATP production and maintain cardiac membrane integrity [46]. CK, which is an enzyme found free in the cytoplasm of myocytes, leaks from these cells when they are damaged and is considered a muscle-specific leakage enzyme. Furthermore, the high levels of serum CK indicate cardiac damage [47]. Cardiac troponins (cTn) are the best markers for the identification of myocardial cell injury because of their superior sensitivity and specificity compared with other biochemical markers [48]. Additionally, cTnT measurements are a specific and sensitive method for the early and late diagnosis of acute myocardial infarction [49]. Serum LDH activity originates from several tissues, including the liver, skeletal muscle, heart muscle, kidney, lung, and erythrocytes [36]. As shown in the present work, remarkable increases in the serum activities of LDH and CK, as well as cTnT concentrations, were observed in groups IV and V. However, a significant increase in LDH and CK levels occurred only in group III. Our data revealed that LPS injections induced harmful heart lesions, as pointed out by elevated levels of LDH, CK, and cTnT. These observations agree with previous reports [50,51]. Ahmed et al. (2020) [52] reported an elevation of serum markers of cardiotoxicity (CK and cTnI) following LPS administration. LDH activity provides additional information that may help explain the activities of more tissue-specific enzymes (i.e., CK).

In this study, histopathological examinations of the lungs revealed pulmonary changes that were mainly inflammatory with the activation of neutrophils and macrophages, as well as pulmonary responses characterized by bronchoconstriction and alveolar collapse [53]. We suggest that airway constriction was induced by alterations in the amounts of intracellular and alveolar surfactants [54] and the release of endogenous mediators [55]. Liver and heart toxicities observed in LPS-induced intoxicated animals may be attributed to the activation of the transcription factor, nuclear factor-kappa B (NF-κB), and toxic mediators generated by activated macrophages: proinflammatory molecules, cytokines, and reactive nitrogen and oxygen species, such as superoxide and nitric oxide radicals [35]. The liver affects the defensive mechanisms that regulate the entry and processing of LPS in the body. The reported lesions in the present work were mainly in the form of inflammatory-type lesions ranging from slight Kupffer cell activation at low doses to hepatocellular necrosis at high doses. The liver also responds to LPS with the production of cytokines along with its role in the clearance of this endotoxins [56], and reactive oxygen intermediates are also released in response to LPS, primarily by Kupffer cells [57], which explains the mechanism through which LPS can induce hepatotoxic effects. As shown in the present work, the hepatocellular necrosis was mainly confined to the portal area (PA). It needs to be taken into consideration that the liver is considered as the final barrier responsible for the clearance of LPS from the blood and prevention of gastrointestinal bacteria and bacterial products (LPS) from entering the systemic circulation [58]. So, the draining of the absorbed LPS was collected in the portal blood flow; thus, it produced its toxic effect directly in the PA. Regarding the heart, it showed myocardial necrosis, which was more obvious in birds treated with a high dose of LPS. This necrosis might have occurred due to the direct and indirect effects of LPS that release many pro- and anti-inflammatory mediators, such as TNF-α and IL-6, resulting in cardiac injury [59]. Venous congestion and volume overload induced by LPS may be the cause of the myocardial hemorrhage noticed in birds treated with higher doses.

Additionally, in this experiment, the serum level of creatinine, as a renal marker, was measured, and the result showed mild increases in its level in groups IV and V. Furthermore, the histopathological renal changes in this study were mild in the form of slight inflammatory cell infiltration in the parenchyma of the kidney at a low dose of LPS to mild tubular basophilia at medium and high doses. The histological changes in this study due to the treatment of birds with different doses of LPS were dose dependent [60,61]. It seems that LPS induced dysfunction of the renal microvascular system through the release of different biologically active mediators responsible for the impairment of renal blood flow [62,63]. Tubular basophilia is considered a feature of renal regenerative changes and tubules lined by cells with basophilic cytoplasm. It occurs after various forms of tubular epithelial cell injury induced by nephrotoxic chemicals [64] or secondary to the vascular damage, probably through ischemia and/or ischemia/reperfusion [65].

## 5. Conclusions

The data presented here provide useful information about serum biomarkers alterations and histopathological changes in turkey poults challenged with graded levels of *E. coli* LPS. This baseline information can be useful for other researchers investigating inflammation or pathophysiological mechanisms in veterinary medicine and in biomedical research. Further future research is suggested to explore more about the cellular and molecular mechanisms underlying these biochemical and histopathological changes following LPS administration in turkey.

## Figures and Tables

**Table 1:** Effect of *E. coli* lipopolysaccharide on the serum biochemical profiles of turkey poults.

Parameters	Control Groups		Treated Groups		
	Group I (NC)	Group II (NS)	Group III (LPS 0.01)	Group IV (LPS 0.1)	Group V (LPS 1)
Triglycerides (mg/dL)	92.00 ± 3.16 ^a^	89.20 ± 6.31 ^a^	86.00 ± 3.90 ^a^	78.50 ± 4.59 ^b^	76.50 ± 7.12 ^b^
Total cholesterol (mg/dL)	132 ± 6.32 ^a^	129 ± 4.52 ^a^	114 ± 4.10 ^b^	101 ± 10.32 ^c^	95 ± 14.24 ^c^
High-density lipoprotein (mg/dL)	85.00 ± 1.90 ^a^	87.67 ± 5.94 ^a^	79.12 ± 3.31 ^ab^	72.50 ± 9.87 ^bc^	69.00 ± 10.95 ^c^
Low-density lipoprotein (mg/dL)	29.00 ± 2.53 ^a^	27.50 ± 2.59 ^a^	19.50 ± 1.97 ^c^	14.00 ± 1.26 ^d^	11.50 ± 0.55 ^e^
Glucose (mg/dL)	293 ± 10.82 ^a^	288 ± 7.54 ^a^	269 ± 9.00 ^b^	271 ± 2.68 ^b^	264 ± 10.21 ^b^
Urea (mg/dL)	2 ± 0.63 ^a^	2 ± 0.00 ^a^	1 ± 0.00 ^b^	1 ± 0.00 ^b^	1 ± 0.00 ^b^
Creatinine (mg/dL)	0.02 ± 0.01 ^c^	0.02 ± 0.01 ^c^	0.02 ± 0.01 ^c^	0.07 ± 0.02 ^b^	0.1 ± 0.00 ^a^
Uric acid (mg/dL)	3.37 ± 0.34 ^a^	3.4 ± 0.22 ^a^	2.63 ± 0.31 ^b^	2.17 ± 0.10 ^c^	1.87 ± 0.18 ^d^
Aspartate transaminase (U/L)	269 ± 9.49 ^d^	276 ± 13.59 ^d^	310 ± 8.94 ^c^	335 ± 8.94 ^b^	353 ± 4.38 ^a^
Alanine transaminase (U/L)	4.00 ± 0.89 ^c^	4.00 ± 0.00 ^c^	5.00 ± 0.89 ^b^	8.00 ± 0.63 ^a^	7.50 ± 0.55 ^a^
Alkaline phosphatase (U/L)	1600 ± 79.06 ^c^	1630 ± 61.67 ^c^	2100 ± 89.44 ^b^	2350 ± 89.44 ^a^	2340 ± 70.11 ^a^
Lactate dehydrogenase (U/L)	985 ± 15.81 ^d^	1040 ± 68.90 ^cd^	1080 ± 27.39 ^bc^	1130 ± 38.82 ^ab^	1170 ± 100.78 ^a^
Creatine kinase CK (U/L)	947 ± 18.97 ^d^	958 ± 24.01 ^d^	1300 ± 89.44 ^c^	1530 ± 81.65 ^b^	1660 ± 21.91 ^a^
Cardiac troponin T (ng/mL)	67.32 ± 8.46 ^c^	60.26 ± 7.47 ^c^	61.46 ± 5.58 ^c^	99.12 ± 8.58 ^b^	198.00 ± 14.30 ^a^

Group I: did not receive any inoculation; Group II: inoculated with the vehicle (normal saline) alone; Group III: inoculated with LPS at 0.01 mg/kg body weight (BW); Group IV: inoculated with lipopolysaccharide (LPS) at 0.1 mg/kg BW; and Group V: inoculated with LPS at 1 mg/kg BW. Data are expressed as means ± standard deviations. Mean values in the same row bearing different superscript letters (a, b, c and d) were significantly different (*p* < 0.05).

**Table 2:** Effect of *E. coli* lipopolysaccharide on the electrophoretic patterns of serum proteins in turkey poults.

Parameters	Control Groups		Treated Groups		
	Group I (NC)	Group II (NS)	Group III (LPS 0.01)	Group IV (LPS 0.1)	Group V (LPS 1)
Total protein (g/L)	3.80 ± 0.09 ^ab^	3.83 ± 0.05 ^ab^	3.77 ± 0.14 ^b^	3.90 ± 0.09 ^a^	3.83 ± 0.06 ^ab^
Albumin (g/L)	1.43 ± 0.05 ^a^	1.37 ± 0.05 ^b^	1.13 ± 0.05 ^c^	1.17 ± 0.05 ^c^	1.17 ± 0.05 ^c^
α1-globulin (g/L)	0.20 ± 0.02 ^c^	0.25 ± 0.05 ^b^	0.19 ± 0.02 ^c^	0.17 ± 0.01 ^c^	0.39 ± 0.05 ^a^
α2-globulin (g/L)	0.94 ± 0.02 ^c^	0.93 ± 0.03 ^c^	1.01 ± 0.02 ^b^	1.19 ± 0.02 ^a^	0.79 ± 0.03 ^d^
β-globulin (g/L)	0.88 ± 0.01 ^bc^	0.90 ± 0.03 ^b^	0.88 ± 0.03 ^bc^	0.84 ± 0.05 ^c^	0.94 ± 0.02 ^a^
γ-globulin (g/L)	0.35 ± 0.01 ^b^	0.39 ± 0.04 ^b^	0.55 ± 0.07 ^a^	0.53 ± 0.11 ^a^	0.55 ± 0.02 ^a^
Albumin/Globulin (ratio)	0.61 ± 0.01 ^a^	0.55 ± 0.03 ^b^	0.43 ± 0.02 ^c^	0.41 ± 0.02 ^c^	0.44 ± 0.02 ^c^

Group I: did not receive any inoculation; Group II: inoculated with the vehicle (normal saline) alone; Group III: inoculated with LPS at 0.01 mg/kg body weight (BW); Group IV: inoculated with lipopolysaccharide (LPS) at 0.1 mg/kg BW; and Group V: inoculated with LPS at 1 mg/kg BW. Data are expressed as means ± standard deviations. Mean values in the same row bearing different superscript letters (a, b, c and d) were significantly different (*p* < 0.05).

## Data Availability

The data supporting the findings of this study are contained within the article.
